# Artificial intelligence-driven clinical decision support systems for early detection and precision therapy in oral cancer: a mini review

**DOI:** 10.3389/froh.2025.1592428

**Published:** 2025-04-28

**Authors:** Manoj Kumar Karuppan Perumal, Remya Rajan Renuka, Suresh Kumar Subbiah, Prabhu Manickam Natarajan

**Affiliations:** ^1^ Centre for Stem Cell Mediated Advanced Research Therapeutics, Saveetha Dental College and Hospitals, Saveetha Institute of Medical and Technical Sciences, Saveetha University, Chennai, Tamil Nadu, India; ^2^ Department of Clinical Sciences, College of Dentistry, Centre of Medical and Bio-Allied Health Sciences and Research, Ajman University, Ajman, United Arab Emirates

**Keywords:** oral cancer, early detection, AI-CDSS, patient data, accurate treatment planning

## Abstract

Oral cancer (OC) is a significant global health burden, with life-saving improvements in survival and outcomes being dependent on early diagnosis and precise treatment planning. However, diagnosis and treatment planning are predicated on the synthesis of complicated information derived from clinical assessment, imaging, histopathology and patient histories. Artificial intelligence-based clinical decision support systems (AI-CDSS) provides a viable solution that can be implemented via advanced methodologies for data analysis, and synthesis for better diagnostic and prognostic evaluation. This review presents AI-CDSS as a promising solution through advanced methodologies for comprehensive data analysis. In addition, it examines current implementations of AI-CDSS that facilitate early OC detection, precise staging, and personalized treatment planning by processing multimodal patient information through machine learning, computer vision, and natural language processing. These systems effectively interpret clinical results, identify critical disease patterns (including clinical stage, site, tumor dimensions, histopathologic grading, and molecular profiles), and construct comprehensive patient profiles. This comprehensive AI-CDSS approach allows for early cancer detection, a reduction in diagnostic delays and improved intervention outcomes. Moreover, the AI-CDSS also optimizes treatment plans on the basis of unique patient parameters, tumor stages and risk factors, providing personalized therapy.

## 1 Introduction

Oral cancer (OC) is becoming an increasingly serious global health challenge; thus, patients must be diagnosed as early as possible to improve survival outcomes. It is the third most common cancer in India and the major cause of cancer in males, with more than 100,000 new cases every year (1–4). The alarming mortality rates associated with OC necessitate intervention strategies focused on early diagnosis, accurate diagnosis, and individualized treatment (5).

OC management is highly complex because of the integration of various information sources, such as clinical examinations, medical imaging, histopathological analyses, and detailed patient history. Conventional methods of diagnosis, including clinical examination and biopsy, offer limited effectiveness in recognizing early lesions and precancerous changes (6–8). These challenges of diagnosis are compounded by these treatments being highly invasive—surgery, chemotherapy, and radiation therapy—on healthy neighboring tissues, thereby lacking accuracy and efficiency (9). This clinical reality necessitates exploration of complementary approaches that can increase early diagnostic accuracy and refine treatment strategies to improve patient outcomes.

The inherent heterogeneity of OC imposes additional complexity on its clinical management. Unlike many other malignant diseases, OC is characterized by the existence of several subtypes arising from aberrations in biological entities such as genes, proteins, RNAs, and metabolites (10). High-level integration of various multiomics data from genomic, transcriptomic, proteomic and metabolomic characterization is needed to characterize these cancers effectively (11–13). With advances in screening methodologies, diagnostic tools, and therapeutic methods with better survival outcomes in recent years, early detection, but still accurate prediction, continues to pose major challenges in OC management (14).

AI-based clinical decision support systems (AI-CDSS) have emerged as novel systems to address these intricate challenges in OC management. These systems use advanced computational techniques such as machine learning, computer vision, and natural language processing to identify subtle patterns and correlations within different types of patient data (15). Therefore, AI methods contribute to early diagnosis, more accurate staging, and the formulation of treatment plans specific to individual patient profiles, tumor biology, and molecular characteristics (16–18). AI-CDSS is particularly well suited for managing the complexities and heterogeneity intrinsic to OC, as it analyzes and merges immense volumes of multimodal data to generate clinically actionable insights.

**Figure 1:**
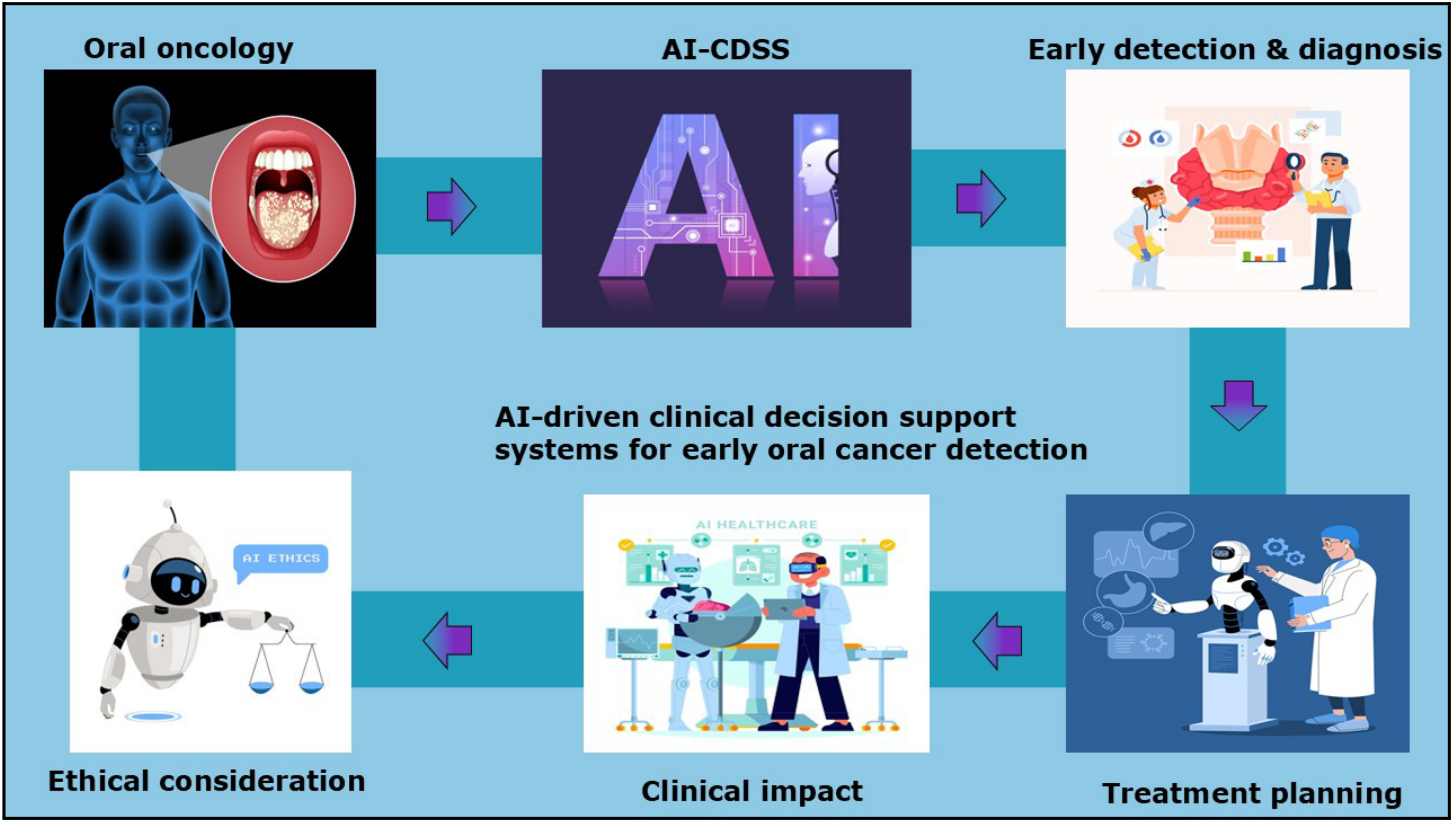
Shows an illustration of AI-driven CDSS for early OC management.

The combination of integrative biology with AI and omics technologies provides opportunities for modeling pathological processes as well as the clinical trajectories of OC (19). Despite growing interest in AI applications for cancer management, there remains a significant knowledge gap regarding the systematic integration of AI-CDSS specifically for OC management. This review highlights the importance of the AI-CDSS approach in early diagnosis, minimization of diagnostic delay, and improvement of opportunities for successful treatment. Additionally, the AI-CDSS can detect tumor stage and risk factors and customize treatment plans on the basis of individual patient parameters, facilitating precision therapy for individual patients. Additionally, the status of the AI-CDSS in OC diagnosis and treatment highlights its role in patient outcome, treatment efficacy, and diagnostic accuracy. Additionally, the limitations and challenges of such systems are discussed, and future work could involve incorporating future technologies into the framework of OC as discussed in Figure 1.

## 2 Role of the AI-CDSS in the early detection and diagnosis of OC

Early detection of OC is critical for improving survival rates and treatment outcomes. Conventional diagnostic methods, such as visual examination and biopsy, on which OC diagnosis relies, these methods are not as effective in detecting early-stage lesions and precancerous conditions (20, 21). Importantly, oral biopsy remains the gold standard for the diagnosis of definite OC and yields critical histopathological evidence that permits precise determination of the type of cancer, grade, and possible molecular characteristics. The AI-CDSS provides a complementary approach to identifying patterns and biomarkers through multimodal analysis of data, including clinical images, patient history, and molecular profiles. One of the prominent applications of AI-CDSS is in the analysis of medical imaging data, although some caution is needed due to current limitations. Preliminary studies have explored the potential of deep learning algorithms in analyzing medical imaging data for the detection of oral precancer lesions and early-stage tumors. However, the present studies are also limited, and stricter validation is needed to prove the accuracy of these tests (22). Notably, among imaging modalities, CT scans are not the most appropriate tool for evaluating soft tissue in oral squamous cell carcinoma (OSCC). However, MRI provides greater soft tissue contrast and is chosen for the delineation of tumor margins, local spread, and lymph node involvement. Furthermore, AI can be employed to identify abnormalities, making earlier detection simpler and enhancing diagnostic potential; however, it cannot be used as a substitute for traditional diagnostic tests.

The AI-CDSS integrates imaging and molecular information, such as age, background, risk factors, and symptoms, with patient data. Large-scale studies of OC diagnosis indicate that AI can be employed to increase the accuracy of diagnosis and support early detection programs. For instance, preliminary findings from trials of experimental deep learning algorithms indicated good performance, with accuracy rates ranging from 81% to 99.7%, sensitivities ranging from 79% to 98.75%, specificities ranging from 82% to 100%, and AUC rates ranging from 79% to 99.5% (23). These studies demonstrate technical feasibility but lack robust prospective validation in diverse clinical environments. In contrast, natural language processing techniques for extracting informative data from unstructured sources of data, such as clinical notes and pathology reports, are also applied in the AI-CDSS (24). It also enables the grading and classification of OC lesions, which is crucial in treatment planning, prognosis prediction, and early detection. A deep learning model was applied to classify OSCC from histopathological images with a 93.2% accuracy rate (25).

Despite these promising developments, the AI-CDSS must remain a decision-support system, thus complementing and augmenting the knowledge of health professionals and not replacing them (26). Additionally, further research and validation studies are needed before the universal application of the AI-CDSS for the diagnosis and management of OC can be achieved. In this manner, issues related to data integrity, algorithmic bias, and the integration of clinical practice should be resolved. The various applications of AI-CDSSs in the management of OC, such as early detection, diagnosis, treatment planning, clinical implementation, ethical and regulatory concerns, challenges, and potential directions, are further discussed in Table 1.

**Table 1:** Application of artificial intelligence in the early detection, diagnosis, and treatment planning of cancer.

Application	Technique	Purpose	Stage	References
Early detection	Deep learning for medical image analysis (CT, MRI, etc.)	Identify abnormalities, precancerous lesions, and early-stage tumors	Experimental	(27)
	Integration of multimodal data (imaging, clinical, molecular)	Comprehensive patient assessment for early detection	Experimental	(28)
	Natural language processing of clinical notes and reports	A Strategy for Deploying Cloud-Based Natural Language Processing Systems for Clinical Text	Emerging/Clinically Validated (Limited)	(29)
	The Effectiveness of Artificial Intelligence in Detection of OC	Early detection and diagnosis of OC using AI techniques	Experimental	(23)
Diagnosis	Deep learning for histopathological image analysis using deep and hybrid learning approaches	Early diagnosis of oral squamous cell carcinoma based on histopathological images	Experimental	(30)
	Combination of optical imaging modalities and AI approaches	Improve early detection and diagnosis of oral and oropharyngeal squamous cell carcinoma (OPSCC)	Experimental	(31)
Treatment planning	Analysis of clinical, genomic, imaging, and multiomics data	Identify deep-level information in genomics, transcriptomics, proteomics, radionics, digital pathological images	Experimental	(32)
	Machine learning for outcome prediction	Predict disease course, survival, and treatment response	Emerging/Clinically Validated (Limited)	(33)
	Integration of high-throughput data and AI techniques	Development of personalized medicine through analysis, integration, and interpretation of massive biomedical data	Experimental	(34)

## 3 Ai models in OC detection and treatment

AI applications in OC management generally involve diverse computational models with separate architectures and abilities for specific aspects of detection and treatment. Understanding the specifics of these models is crucial in evaluating their clinical use and limitations. Table 2 provides a comprehensive overview of various AI techniques and their specific applications in OC detection.

**Table 2:** Ai techniques and their applications in OC detection.

AI technique	Method/Algorithm	Application in OC	Performance metrics	Key advantages	References
Deep learning	Convolutional Neural Networks (CNN)	Analysis of oral cavity images for lesion detection	Accuracy: 86%–97%, Sensitivity: 85%–96%,	Automated feature extraction from complex visual data; ability to detect subtle patterns invisible to human eye	(47)
	Recurrent Neural Networks (RNN)	Prediction of cancer progression from temporal data	AUC: 0.82–0.91	Ability to analyze sequential medical data and capture temporal dependencies	(48)
	Generative Adversarial Networks (GAN)	Data augmentation for limited histopathological datasets	Improved model accuracy by 8%–15%	Addresses data scarcity through synthetic image generation	(49)
Machine learning	Random Forest	Risk stratification based on clinical variables	Accuracy: 78%–89%, F1-score: 0.75–0.86	Robust against overfitting; handles heterogeneous data types	(50)
	Support Vector Machines	Classification of lesion types from spectroscopic data	Sensitivity: 81%–92%, Specificity: 83%–94%	Effective with high-dimensional data; robust classification boundaries	(51)
Computer vision	Object Detection (YOLO, Faster R-CNN)	Automated detection of suspicious regions in oral cavity images	Precision: 83%–94%, Recall: 81%–92%	Real-time analysis; can be deployed on mobile devices for screening	(52)
	Semantic Segmentation	Precise delineation of tumor margins from imaging data	Dice coefficient: 0.79–0.88	Assists surgical planning; improves resection accuracy	(53)
	Feature tracking algorithms	Monitoring lesion changes over time	Detection of 7%–12% more progression cases than manual review	Early identification of malignant transformation in precancerous lesions	(54)
Multimodal integration	Ensemble methods	Integration of clinical, imaging, and -omics data	Improved predictive accuracy by 12%–18% over single modality approaches	Comprehensive patient profiling; captures multidimensional disease patterns	(55)
	Graph neural networks	Modeling relationships between biological pathways and clinical manifestations	AUC: 0.85–0.93	Captures complex relationships in heterogeneous data	(56)

### 3.1 Deep learning architectures

Deep learning models, such as convolutional neural networks (CNNs) are the foundation for the detection of OC in images. They have a definite structure that enables them to distinguish visual patterns. CNNs extract visual patterns through a layered structure: initial layers detect simple features like edges and textures, while later layers identify complex patterns specific to malignant transformations (35). Popular CNNs like ResNet and Inception-v3 are utilised in histopathological image analysis for OC (36). It is also valuable for transfer learning, where islands of networks that had been previously trained on ImageNet trained further on smaller, oral-cancer-specific datasets have shown advantages due to the scarcity of available annotated OC images (37). For example, a modified ResNet-50 architecture was used by Aubreville et al. to analyze tissue in oral epithelium samples with an accuracy of 93.1% in separating normal from cancerous tissue, while attention maps highlighted diagnostically relevant regions corresponding with pathologist annotations (38).

U-Net architectures designed for biomedical images have shown essential accuracy in tracing tumor borders on OC images. This kind of network adopts an encoder‒decoder layout with skip connections that retain spatial information essential for precise segmentation (39). Kanakarajan et al. reported that their modified U-Net architecture for contrast-enhanced MRI images achieved a mean Dice coefficient of 0.87 for tumor segmentation, with an accompanying reduction in interobserver variability (40). This method allows precise segmentation that can help clinicians in decision-making regarding surgical planning and radiation therapy targeting.

Long short-term memory (LSTM) networks, which are arch species of Recurrent Neural Networks (RNNs), have made crucial marks in the area of temporal data analysis in OC progression, as well as treatment response monitoring. These architectures integrate memory cells capable of capturing long-range dependences in sequential data, making them feasible for the study of time series clinical measurements and longitudinal imaging (41). Thus, the LSTM networks have been applied to the prediction of disease recurrence, basing the model on temporal arrangements of biomarkers in the posttreatment period and yielding accuracies ranging from 83% to 89% greater than those of standard clinical assessment techniques.

### 3.2 Classical machine learning models

Random forest methods show immense promise in predicting the risk stratification and prognosis of OC. These methods generate a multitude of decision trees by training them on bootstrapped data samples and randomly selected feature subsets, aggregating their predictions to reduce overfitting and increase generalization (42). For instance, De Silva et al. developed a random forest-based model using a combination of clinical variables, histopathological features, and selected biomarkers, achieving 84.6% accuracy in predicting lymph node metastasis in patients with OSCC (43, 44). The random forest models provide inherent feature importance rankings, offering clinically interpretable evidence that perineural invasion and tumor budding are particularly significant predictors.

### 3.3 Multimodal fusion architectures

Multimodal fusion networks utilize dedicated subnetworks per data modality (imaging, genomic, or clinical) prior to fusing their features in layers. These structures use attention mechanisms to learn to adaptively weight the relevance of various modalities on a case-by-case basis (45). In OC, this strategy has been shown to outperform single-modality models by drawing from complementary information from different clinical exams, histopathology, radiomics, and genomic markers. Recent applications have achieved a 5%–10% improvement in predictive accuracy over single-modality methods through learned cross-modal attention, which simulates the integrative diagnostic process of multidisciplinary tumor boards (46).

## 4 AI-CDSS in treatment planning for OC

Personalized treatment plans customized by tumor biology, molecular signatures, and patient features are referred to as precision oncology. The AI-CDSS is among the most significant technologies that allow precision medicine in OC by combining multiple data sources to support personalized treatment. AI algorithms can analyze clinical, genomic, imaging, and other data to detect patterns and biomarkers suitable for therapeutic interventions in individual patients.

The AI-CDSS represents a significant advance in surgical planning and treatment intervention optimization. The systems are intended to support medical professionals by integrating a wide range of data sources, thus enabling more comprehensive and tailored treatment advice. The AI-CDSS supported improved surgical decision-making by deciphering complex patient information, such as clinical, imaging, and molecular data. Important applications for artificial intelligence technologies in surgical planning include improvements in tumor margin delineation, the construction of three-dimensional tumor models, and the prediction of possible surgical complications (57). Machine learning algorithms analyze patient-specific information and classify those risks to identify the best surgical strategies that can enhance outcomes and ensure the safety of patients in surgical operations (58). The evolution of genomic and molecular profiling has revealed the heterogeneity of OC and the promise of targeted therapy. The AI-CDSS strategy is intended to help surgeons make data-driven recommendations that can be utilized to plan surgeries. The prediction of likely surgical complications, determination of the prominent intervention, and minimization of invasiveness guarantee the successful resection of tumors (59). The AI-CDSS also integrates information from several omics platforms, such as genomics, transcriptomics, proteomics and metabolomics, to develop an integrated molecular profile of oral tumors. Multiomics models yield rich information on the complexity of individual patient cancer profiles, enabling precise targeting of the main processes and pathways for personalized treatment strategies (60).

In addition to surgical intervention, the AI-CDSS can be utilized to enhance other treatment protocols. These options range from radiation planning therapy to the administration of chemotherapy, as well as combination regimen planning. Such platforms can review immense patient information, thereby generating highly personalized treatment strategies on the basis of the individual characteristics of the patients, tumor biology, and projected results of treatment (61). The AI-CDSS can also be extended to postsurgical management and follow-up. Machine learning algorithms may help monitor patient recovery, predict potential recurrence, and provide support for long-term treatment strategies. However, it is very important to emphasize that these technologies are designed to support, not replace, the clinical expertise of healthcare professionals.

### 4.1 Clinical impact of the AI-CDSS in OC management

The integration of the AI-CDSS into healthcare is important for establishing clinical benefits, enhancing clinical decision-making, increasing diagnostic accuracy, and personalizing treatment plans (62). However, their use needs careful clinical validation and an understanding of physician perceptions, which are important for the successful implementation of technologies such as CURATE, an AI-driven personalized dosing CDSS. Physicians play an important role in implementing new clinical technology because developers are provided with growth as well as feedback from patients. In order to facilitate the clinical validation, integration, and eventual adoption of the CURATE AI-CDSS, it is essential to understand the perspectives of physicians (63). In addition to performance evaluations, clinical validation is needed as a multidimensional approach. The comparison of the AI-CDSS with expert clinician diagnoses should be performed prospectively and through randomized controlled trials across various clinical settings, and the methodological protocols need to be standardized. These studies should also incorporate comprehensive evaluation metrics that extend beyond diagnostic accuracy, including clinical relevance, cost-effectiveness, and enhancements in patient care pathways (64). The key performance indicators should be sensitivity, specificity, positive and negative predictive values, and clinical impact measures such as a reduction in diagnostic delays, improvements in treatment planning precision, and long-term patient outcomes. Validation studies must involve diverse patient cohorts with different demographic, genetic, and socioeconomic backgrounds to establish the reliability and generalizability of the AI-CDSS (65).

### 4.2 AI/ML in head and neck cancer diagnosis

AI and machine learning approaches are gradually becoming very important in automated image analysis for diagnosis of patients suffering from head and neck cancers (HNC), particularly OC. Recent reviews, based on analysis of 32 studies, show that traditional machine learning methods were used in 29 studies, whereas deep learning (DL) was used in 25, with only 6% of combined use (66). Such AI/ML models demonstrated improved performance compared with human evaluation in detecting HNC-enhancing diagnostic accuracy and thus have immense potential. However, their clinical applicability requires further validation before they can be accepted as reliable and useful.

A significant limitation is the low generalizability of AI models across different populations and environments. Models developed using particular datasets, e.g., fair-skinned populations, tend to underperform in darker-skinned populations, failing to fetch essential presentations of oral lesions (94). This limitation is further deteriorated by etiological variations across regions. For instance, betel quid chewing is a leading risk factor in South Asia, whereas tobacco and alcohol consumption are more prevalent in Western nations (95). Consequently, an AI model trained in one region may not generalize well to another, which underscores the importance of having diverse training datasets that capture global differences in disease presentation and risk factors. Furthermore, the technical inconsistencies develop increasingly complicated designs for model implementation. Variations in imaging modalities (e.g., CT, MRI), device calibration, and histopathological staining protocols affect the performance of AI models when applied in different clinical settings (96). Normally, MRI contrast or staining techniques altered the appearance between the tumors and lesions, leading to a class misrepresentation of the AI model.

Most of the AI studies are retrospective, and this bias makes them overestimate performance from what is observed in the controlled settings of research in comparison to their realities (97). Only a few prospective clinical studies have been conducted, and their lack makes it difficult to understand the varied clinical conditions under which these models perform. Systematic validation frameworks with multiphase clinical trials are now a pressing need to close that gap. They should involve not only algorithm accuracy tests but real-world clinical utility, patient outcomes, and impacts on treatment strategies. The primary endpoints of efficacy include but are not limited to early detection rates, diagnostic precision, treatment optimization, and patient survival (67). Preliminary studies have reported high accuracy rates (81%–99.7%), but systematic validation frameworks involving multiphase clinical trials are urgently needed. These clinical trials should evaluate not only the accuracy of algorithms but also their real-world clinical utility, patient outcomes, and impacts on treatment strategies. Early detection rates, diagnostic accuracy, treatment optimization, and patient survival are critical benchmarks for assessing AI-CDSS effectiveness (67).

### 4.3 AI-CDSS for OC screening and early detection

**Figure 2:**
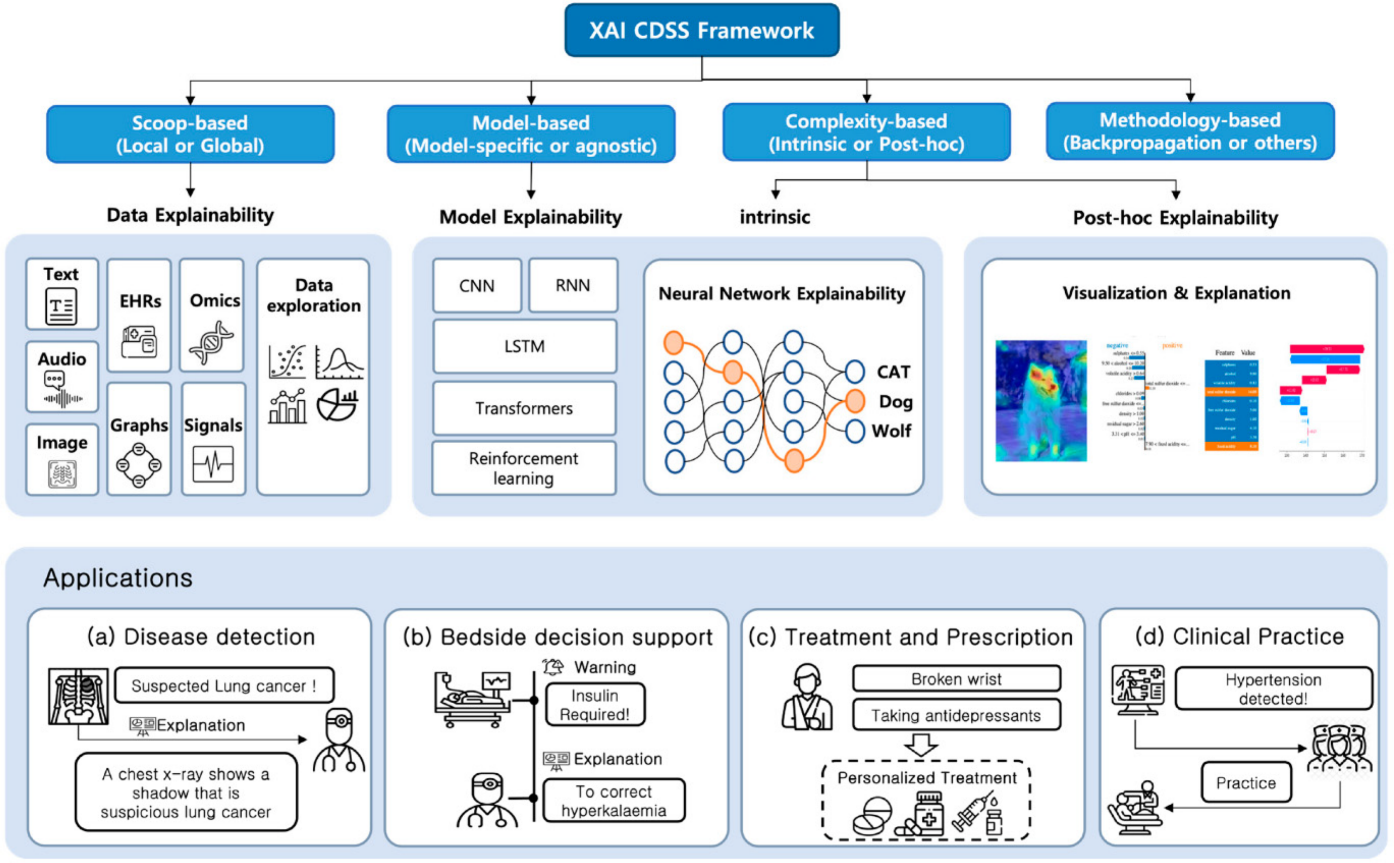
Explainable AI (XAI) clinical decision support systems framework for healthcare applications. The framework illustrates four main approaches to XAI CDSS: Scoop-based, Model-based, Complexity-based, and Methodology-based. Each approach offers different explainability methods including data explainability (using text, EHRs, omics, audio, images, graphs, and signals), model explainability (through CNN, RNN, LSTM, transformers, and reinforcement learning), intrinsic explainability using neural networks, and *post-hoc* explainability through visualizations. The bottom panel demonstrates practical applications in **(A)** disease detection, **(B)** bedside decision support, **(C)** treatment and prescription, and **(D)** clinical practice implementation. Adapted from Kim et al. (70).

The incidence of OC and potentially malignant disorders is increasing, and mortality rates are greater due to limited access to resources for early detection. The use of AI-CDSS in smartphone-captured images of the oral cavity via deep learning models is highly promising. These methods have shown promising results in differentiating between suspicious and nonsuspicious oral lesions, hence aiding in the screening and early detection of OC (68). Despite the rapid development of AI-CDSS, how these systems are being adopted is still unknown. According to a recent study on Chinese hospitals, 23.75% have adopted the AI-CDSS. Challenges included methodological biases, data quality issues, and a lack of improvements in functions. Furthermore, respondents from hospitals that have already implemented the AI-CDSS expressed varying levels of satisfaction, with responses ranging from “neutral” to “satisfied” (69). The complexity of AI-CDSS implementation requires frameworks that balance technical sophistication with clinical interpretability. Figure 2 illustrates a comprehensive Explainable AI (XAI) CDSS framework that outlines different approaches to achieving transparency and interpretability in healthcare AI systems. This framework is particularly relevant for OC management, where clinician trust and understanding of AI recommendations are essential for the successful integration of AI into practice. To ensure that the clinical impact of the AI-CDSS in the management of OC is maximized, research should focus on systematic clinical validation frameworks that include comprehensive prospective studies about the performance of the AI-CDSS compared with the gold-standard clinical assessments. The resolution of issues, including methodological biases, data quality, and system functionality, may significantly enhance diagnostic accuracy, optimize treatment strategies, and improve patient outcomes in OC.

### 4.4 AI in multiomics data integration for OC

The integration of multiomics data presents a unique new opportunity for AI applications in OC management. These AI approaches are remarkably versatile in handling heterogeneous datasets because they involve the application of multiple advanced mechanisms. AI-based integration of heterogeneous data can utilize deep learning architectures, particularly multimodal neural networks that accept data types of various natures as inputs, enabling their original form and characteristics to remain (71). These networks use a particular encoding layer for each data modality—a convolutional layer for imaging data and recurrent networks for sequential genomic data—and they feed attention mechanisms on clinical variables. Integrative transfer learning exploits a high-dimensional data approach in pretraining models over a large dataset before fine-tuning on OC data, thereby effectively retaining important biological signals shiftable in smaller cohorts, which would not have previously been used for fine-tuning (72). More recent methods include graph neuro-networks that consider interrelationships of multiomics as interlinked nodes; therefore, this also enables the identification of complex cross-platform biomarker patterns (73). For instance, it can link specific genomic abnormalities with resulting changes in protein expression and associated radiographic features in oral tumors, achieving molecular phenotyping on a holistic basis that exceeds the explanatory power of single-modality approaches (74).

Despite these developments, the integration of multiomics remains a challenge. Data harmonization is exceedingly difficult, particularly when examining data obtained from two different platforms or protocols in diverse institutions. AI methodologies, such as domain adaptation and generative adversarial networks, can provide promising solutions for eliminating batch effects and protocol variations without altering the biological signal characteristics of the data (75, 76). Missing data are a significant problem for multiomics studies, and current methods for imputing such missing data are becoming increasingly sophisticated because they consider correlations across platforms to estimate missing values more accurately than traditional statistical methods (77). Another continuing challenge concerns the computational efficiency of processing multidimensional high-omics datasets that often require large amounts of computing resources, which may not be available in a clinical setting. Model compression and edge computing developments are beginning to help fill this implementation gap (78). The federated learning approach provides a valuable opportunity for collaborative AI model development across multiple institutions, enabling the creation of robust multiomics integration models for diverse patient populations while safeguarding sensitive patient data and addressing privacy concerns (79).

OC specifically benefits from AI multiomics integration, which provides an essential opportunity for characterizing the molecular heterogeneity of malignancies. Recent studies have employed these methods to identify molecular subtypes with distinct prognoses and treatment responses not discovered by classical histopathological classification (80). Clarifying the relationships between genomic alterations and protein expression patterns has led to the identification of novel druggable targets in certain cases of resistant OC. Most importantly, new trends could also allow the integration of spatial transcriptomics and digital pathology with traditional omics datasets, thereby providing insight into the tumor microenvironment and cellular interactions that drive OC progression (81). These comprehensive strategies are enhancing a pivotal shift toward AI-driven multiomics integration in the interrogation of OC biology, thus enabling more precise diagnostic and therapeutic interventions specific to an individual's molecular profile.

## 5 Ethical considerations and regulatory aspects

Clinical decision support systems, or AI-CDSSs, have demonstrated promise in preclinical testing, although there is currently insufficient evidence of their advantages in real patient care. The DECIDE-AI reporting guidelines were developed to improve standards for early clinical evaluation of AI systems in an attempt to address this gap. In pursuit of transparency, security, and reproducibility of outcomes, these requirements include both specific and general reporting components (82). Despite a particular focus on global health issues and crucial ethical, legal, and social ramifications (ELSIs), healthcare has embraced AI integration in increasing numbers. The effects of AI on patient‒physician relationships, accountability, regulatory frameworks, algorithmic transparency, and patient safety are among the main issues. Even if AI has the ability to improve patient care, it is still vital to address ELSI challenges, especially in regard to the evolving dynamics of patient‒physician interactions (83). The AI-CDSS has the potential to improve the quality and reach of OC screenings. However, addressing inequality in power between healthcare personnel and AI systems is necessary for successful integration. The development of AI-based healthcare decision-making involves the development of mutually beneficial connections between physicians and AI to maintain public acceptability and avoid ethical issues (84).

### 5.1 Regulatory framework and clinical guideline integration for AI-CDSS in OC

**Figure 3:**
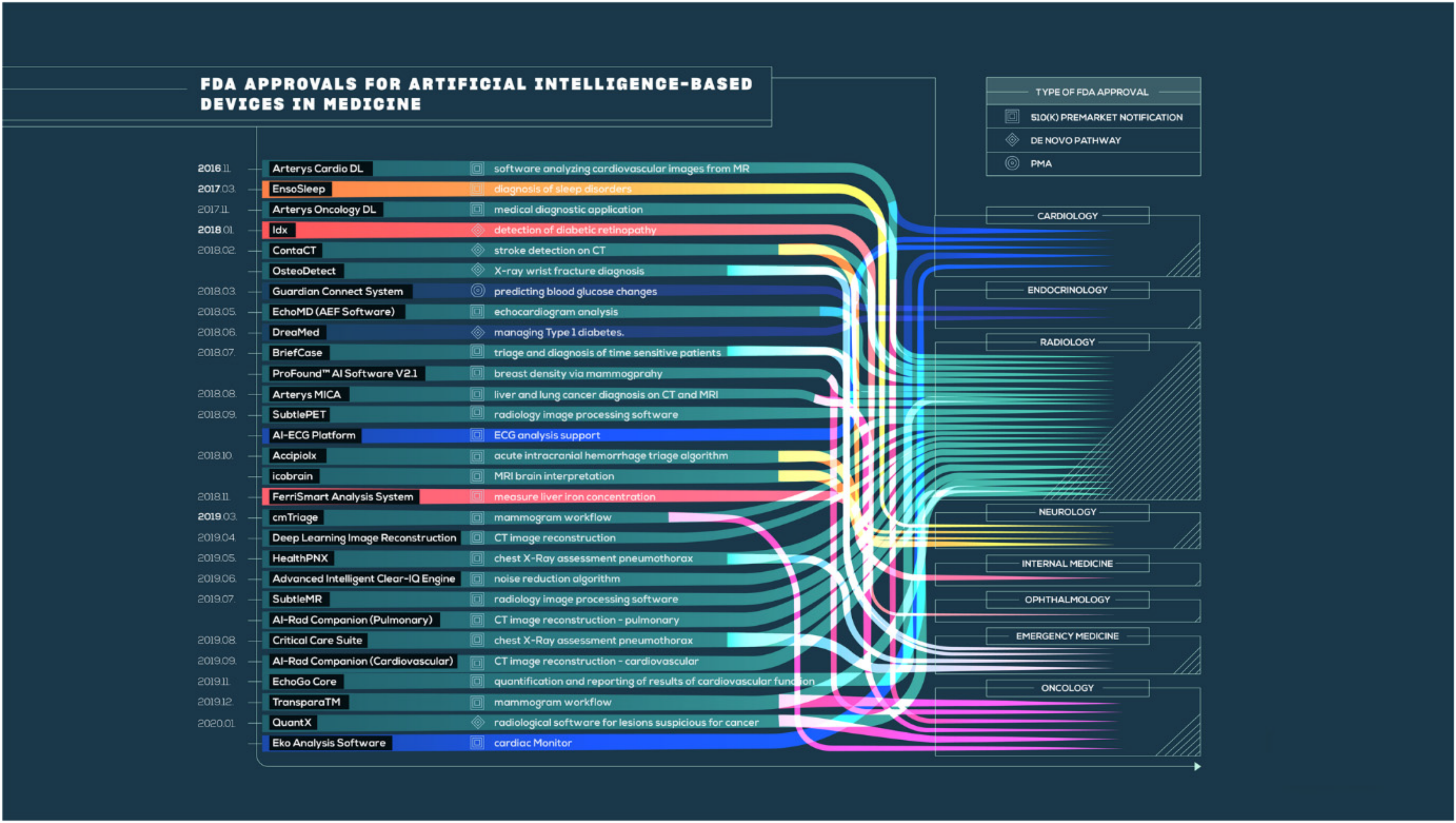
FDA approvals for AI-based medical devices across specialties. Adapted from Benjamens et al. (86).

The AI-CDSS needs to be integrated into existing clinical workflows without a detailed examination of existing regulatory frameworks to align with clinical practice guidelines (85). Most of the world's regulatory bodies are trying to develop strategies for evaluating and approving AI-based medical technology (Figure 3), but there are still major gaps in the application of these strategies for OC treatment. For example, the United States FDA's establishment of a regulatory framework is based on risk for AI/ML-based Software as a Medical Device (SaMD) under its Digital Health Center of Excellence; a proposed regulatory framework for the modification of AI/ML-based SaMD that recognizes the unique “learning” capability of these systems (86). However, most AI-CDSS developed for OC are being researched, with no actual regulatory approval for clinical deployment. This poses a crucial challenge for healthcare institutions, which are inclined to consider the adoption of such systems. The European Union's Medical Device Regulation (MDR) and *in vitro* Diagnostic Regulation (IVDR) categorize the majority of AI-CDSS as Class IIa or above medical devices, which necessitate notified body conformity assessment and substantial clinical evidence (87). These demands pose a major threat to the translation of promising studies into clinical tools. Regulatory agencies are challenged by the difficulty of balancing innovation and patient safety. The “black box” complexity of most deep learning algorithms makes conventional evaluation strategies based on knowledge of the exact mechanism of action difficult. Regulators need to find new ways of validating systems that learn continuously and whose performance could shift over time following initial approval (88).

The disconnect between rapidly advancing AI technologies and more revised clinical guidelines makes clinicians and institutions uncertain. Due to the absence of clear guidelines on how insights from AI-CDSS should influence clinical decision-making, practitioners currently do not have a standardized protocol for resolving potential conflicts between algorithmic recommendations and traditional clinical judgment (89). The lack of formal integration channels complicates routine AI-CDSS adoption despite encouraging technical performance. Future regulatory approaches for successful clinical integration of the AI-CDSS should ensure the rigorous validation of practical implementation pathways. Such standards provide clinical performance evaluation and technical accuracy to measure actual outcomes and qualities of care. Moreover, regulatory frameworks should address sustaining performance over time in patient populations and clinical settings concerning the continued availability of monitoring requirements (12).

## 6 Critical limitations of the AI-CDSS in clinical implementation

AI-CDSS offer promising potential for improving OC management, yet their clinical implementation faces significant limitations and challenges. However, many major limitations need to be addressed before these systems can be adopted clinically on a wide scale. These challenges are technical, clinical, and implementation types that come into being, and all affect the real utility of the systems in the long run (90). OC is a complex illness, and it may not always be possible to obtain the clinical, imaging, and molecular data required to train accurate AI models, especially with limited resources. The development of comprehensive multimodal datasets can be a challenging process that requires significant effort. Standardizing data collection procedures and ensuring data security and privacy are also crucial.

The primary limitations in the development of robust AI models for OC necessitate large, heterogeneous, high-quality datasets that are mostly unavailable in clinical settings (23, 91). This data scarcity creates significant challenges, including poor demographic representation, inconsistent annotation quality, and integration incompatibility across multiple modality datasets. Available datasets often contain insufficient diversity across age, ethnicity, genetic background, and socioeconomic factors, resulting in models derived from homogeneous populations being unsuitable for clinical applications (92). The process of expert annotation for imaging and histopathology inherently introduces random variability and potential errors; interobserver variability among pathologists propagating into AI training data subsequently results in inconsistent performance of the model (93). Furthermore, combining heterogeneous data types (imaging, genomic, and clinical) requires sophisticated data techniques that are still evolving, whereas differences in data collection protocols across institutions further complicate integration efforts. Recent studies mostly refer to technical performance metrics, such as accuracy and AUC, rather than useful clinically relevant outcomes, mostly in terms of survival, morbidity, or quality of life (98). Only a few randomized controlled trials have compared the AI-CDSS with standard practice. These limitations indicate that the findings of AI-CDSS in OC management should be interpreted with significant attention.

## 7 Conclusion

Artificial intelligence-based clinical decision support systems (AI-CDSS) hold significant promise to enhance OC through early diagnosis, improved diagnostic accuracy, and individualized treatment planning. AI-CDSS can interpret various types of data with advanced machine learning and deep learning algorithms. However, despite their technical promise, AI-CDSS for OC remains experimental, and clinical validation and real-world utility have yet to be established. Significant challenges include insufficient validation, limited high-quality datasets, algorithm bias, standardization issues, regulatory uncertainties, and limited generalizability across diverse populations (e.g., regional risk factors and ethnic differences). In order to advance AI-CDSS towards clinical applications, future advancements require focus on interdisciplinary collaboration between clinicians, data scientists, and ethicists to develop systems that are technically robust, clinically meaningful, and ethically implemented. Addressing these limitations is essential to ensure AI-CDSS is adopted into clinical practice and fulfills its transformative potential in OC management.
